# Supplementary material for: Identification of photoperiod-induced specific miRNAs in the adrenal glands of Sunite sheep (Ovis aries)
Source: Front Vet Sci. 2022 Jul 22;9:888207. doi: 10.3389/fvets.2022.888207 (PMC9354845; doi:10.3389/fvets.2022.888207)
Supplement: Supplementary file 1 [file Data_Sheet_1.ZIP › Supplementary file/miRDeep2 file.zip!/miRDeep2 file/Novel_Structure/SPLP42A2/Novel_639.pdf]

| | | |
|---|---|---|
| Provisional ID | : | NC_019484.2_43479 |
| Score total | : | 2.7 |
| Score for star read(s) | : | -1.3 |
| Score for read counts | : | 0 |
| Score for mfe | : | 2.4 |
| Score for randfold | : | 1.6 |
| Score for cons. seed | : | |
| Total read count | : | 30 |
| Mature read count | : | 24 |
| Loop read count | : | 0 |
| Star read count | : | 6 |

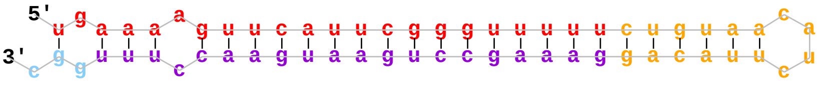


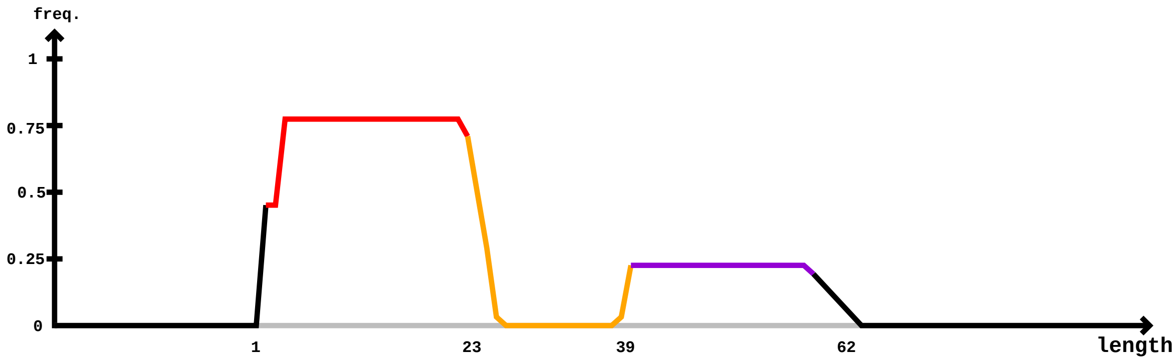


**Mature** **Star**

```
5'- uccccacauauuggguuggcugaaaaguucauucggguuuuucuguaacaucuuacaggaaagccugaaugaaccuuuggccaacccaauauuuccgaccaguugcaagggc -3'    obs
    uccccacauauuggguuggcugaaaaguucauucggguuuuucuguaacaucuuacaggaaagccugaaugaaccuuuggccaacccaauauuuccgaccaguugcaagggc         exp
    .......(((((((((((((((((..((((((((((((((((((((((....)))))))))))))))))))))).)))))))))))))))))......((........))..    reads    mm    sample
    ....................ugaaaaguucauucggguuuu.......................................................................    2        0     seq
    ....................ugaaaaguucauucggguuuuu......................................................................    12       0     seq
    ......................aaaGguucauucggguuuuuc.....................................................................    1        1     seq
    ......................aaaGguucauucggguuuuucu....................................................................    1        1     seq
    ......................aaaaguucauuAggguuuuucu....................................................................    7        1     seq
    ......................aaaaguucauuUggguuuuucug...................................................................    1        1     seq
    .........................................................ggaaaAccugaaugaaccuu...................................    1        1     seq
    ..........................................................gaaaAccugaaugaaccuuu..................................    6        1     seq
```
